# Radiomic Analysis of Striatal [^18^F]FDOPA PET Imaging in Patients with Psychosis for the Identification of Antipsychotic Response

**DOI:** 10.1007/s11307-025-02014-3

**Published:** 2025-05-05

**Authors:** Astrid Schiulaz, Giovanna Nordio, Alessio Giacomel, Rubaida Easmin, Andrea Bettinelli, Pierluigi Selvaggi, Steven Williams, Federico Turkheimer, Sameer Jauhar, Oliver Howes, Mattia Veronese, FDOPA PET Imaging Working Group, Ilinca Angelescu, Micheal Bloomfield, Ilaria Bonoldi, Faith Borgan, Tarik Dahoun, Enrico D’Ambrosio, Arsime Demjaha, Jecek Donocik, Alice Egerton, Stephen Kaar, Euitae Kim, Seoyoung Kim, James Maccabe, Julian Matthews, Robert McCutcheon, Philip McGuire, Chiara Nosarti, Matthew Nour, Maria Rogdaki, Grazia Rutigliano, Peter S. Talbot, Luke Vano

**Affiliations:** 1 Department of Information Engineering, University of Padua, Padua, Italy; 2 Department of Neuroimaging, Institute of Psychiatry, Psychology & Neuroscience, King’s College London, London, UK; 3 Medical Physics Department, Veneto Institute of Oncology IOV - IRCCS, Padova, Italy; 4 Department of Translational Biomedicine and Neuroscience, University of Bari Aldo Moro, Bari, Italy; 5 Department of Psychosis Studies, Institute of Psychiatry, Psychology & Neuroscience, King’s College London, London, UK; 6 Psychiatric Imaging Group, MRC London Institute of Medical Sciences, Hammersmith Hospital, Imperial College London, London, UK; 7 South London and Maudsley NHS Foundation Trust, London, UK

**Keywords:** PET, FDOPA, Radiomics, Schizophrenia

## Abstract

**Purpose:**

Schizophrenia (SCZ) is a severe psychiatric disorder marked by abnormal dopamine synthesis, measurable through [^18^F]FDOPA PET imaging. This imaging technique has been proposed as a biomarker for treatment stratification in SCZ, where one-third of patients respond poorly to standard antipsychotics. This study explores the use of radiomics on [^18^F]FDOPA PET data to examine dopamine synthesis in SCZ and predict antipsychotic response.

**Methods:**

We analysed 273 [^18^F]FDOPA PET scans from healthy controls (*n* = 138) and SCZ patients (*n* = 135) from multiple cohorts, including first-episode psychosis cases. Radiomic features from striatal regions were extracted using the MIRP Python package. Reproducibility was assessed with test–retest scans, selecting features with an intraclass correlation coefficient (ICC) > 0.80. These features were grouped via hierarchical clustering based on Spearman correlation. Regression analysis evaluated sex and age effects on radiomic features. Predictive power for treatment response was tested and compared to standard imaging analysis obtained from the Standardised Uptake Value ratio (SUVr) of striatal over cerebellar tracer activity.

**Results:**

Out of 177 features, 15 met the ICC criteria (ICC: 0.81–0.99). Age and sex influenced features in patients but not in controls. The best performance were was by the GLCM joint maximum feature, which effectively differentiated responders from non-responders (AUC: 0.66–0.87), but did not reach statistical significance in classification over SUVr.

**Conclusion:**

Radiomic analysis of [^18^F]FDOPA PET supports its use as a biomarker for assessing antipsychotic efficacy in schizophrenia, highlighting differential striatal tracer uptake based on patient response. While it provides modest classification improvements over standard imaging, further validation in larger datasets and integration with multivariate classification algorithms are needed.

**Supplementary Information:**

The online version contains supplementary material available at 10.1007/s11307-025-02014-3.

## Introduction

Schizophrenia (SCZ) and related psychotic disorders contribute to global disability, affecting around 24 million people worldwide [1]. Alteration of the dopaminergic system plays a crucial role in the pathophysiology of the disorder [2, 3]. Increased striatal dopamine synthesis capacity is a consistent finding in patients with SCZ and across psychotic disorders [3–8]. Antipsychotic medications counteract this dopamine elevation, principally by blocking dopamine D2 receptors in the striatum. However, only two-third of patients respond to first-line antipsychotic medications (hereafter *responders*). The remaining one-third (hereafter *non-responders*) may be considered as having treatment resistant illness for which the recommended treatment is clozapine [9]. Non-response may be present from illness onset, in around one fifth of people with SCZ [10]. However, antipsychotic non-response can currently only be determined through failed treatment trials, as there are no biomarkers available to predict treatment response from illness onset.

PET imaging in combination with [^18^F]FDOPA has been recently proposed as a potential biomarker for predicting treatment response in psychosis [11]. Crucially, striatal dopamine synthesis capacity, as measured by [^18^F]FDOPA PET, has been shown to be elevated at group level in SCZ [12] and in people at clinical high-risk for psychosis (CHR), who subsequently transition to psychotic disorders [13–15]. In contrast, previous studies using [^18^F]FDOPA PET indicated that *non-responders* do not present such elevations [16], thus suggesting that this imaging method may distinguish between responders and non-responders and could potentially be used for early stratification [11].

Current attempts at treatment classification using [^18^F]FDOPA PET in psychosis primarily rely on quantifying the mean striatal signal, either from static or dynamic imaging [11]. However, the heterogeneous tracer distribution in striatal regions, together with a well-defined topology of the dopamine circuits, suggest that methods with higher sensitivity and anatomical precision might increase the discriminant power in separating responders vs non-responders. In addition, current pathophysiological models suggest that dopamine alterations in SCZ are mainly localized to the associative striatum and related circuits [17], thus highlighting the need for more precise methods to capture and quantify morphological and texture abnormalities within the striatum [18, 19].

Radiomics is an emerging field that allows the non-invasive, high-throughput extraction of quantitative metrics, called radiomic features, from medical images. These features capture tissue characteristics (pathological and non-pathological) and can be used to support clinical diagnosis. In addition, the use of radiomics on large datasets has the potential to define new biomarkers that can help understand disease trajectories and support treatment planning [20]. Radiomics, in combination with molecular imaging, has been extensively used in oncology to assess tumour phenotypes (e.g., to characterise tumour morphology and heterogeneity) and monitor longitudinal changes [21]. In this context, radiomics has been applied to [^18^F]FDOPA PET imaging data for the non-invasive prediction of glioma molecular parameters [22], as well as to build prognostic tool in patients affected by high-risk neuroblastoma [23]. While some radiomic studies have been conducted in SCZ [24–26], none have focused on dopaminergic synthesis nor on the striatum.

In this work, we investigated the potential of radiomic methodology applied to [^18^F]FDOPA PET imaging data to explore striatal alterations in dopamine synthesis capacity in patients with SCZ. Our aim was to gain deeper insights into the underlying biology of the disorder [27] and improve the prediction of treatment response using [^18^F]FDOPA PET. By leveraging independent test–retest [^18^F]FDOPA PET datasets from both patients with psychosis and healthy controls, we identified replicable radiomic features and explored their associations with individual demographic variables. We tested the hypothesis that radiomic features could provide a clinically viable framework for predicting treatment response and enhance classification performance beyond that achieved with standard static [^18^F]FDOPA PET imaging analysis.

## Methods

### [^18^F]FDOPA PET data

The [^18^F]FDOPA PET imaging data used in this study were retrieved from the PET imaging data repository available at the Institute of Psychiatry Psychology and Neuroscience (IoPPN) at King’s College London [28].

A total of 273 [^18^F]FDOPA PET scans were used, which comprised 138 healthy controls (80 males, 58 females, age 28.5 ± 7.9 years) and 135 patients with a psychotic disorder according to ICD-10 and DSM criteria [29] (98 males and 37 females, age 32.0 ± 10.8 years). Data were retrieved from several different studies, collected with the following PET tomographs: Hi-Rez Biograph 6 (*N* = 118), Biograph 40 Truepoint (*N* = 50), Biograph Truepoint 6 (*N* = 35), ECAR HR + 962 (*N* = 36), ECAT EXACT 3D (*N* = 7), Biograph TruePoint TrueV (*N* = 27). Among the patient group, 71 were labelled as responders to standard antipsychotic treatment and 64 were classified as non-responders. This classification of patients response followed the clinical criteria as stated in the in the original studies [30–33]. A subgroup of these patients was further reorganised in three different clinical cohorts, representing different stages of the disorder. FDOPA_01 comprises first-episode psychosis (FEP) patients who were recruited if they had a diagnosis of psychotic disorder according to ICD 10 criteria in first episode of the illness [30]. At the time of [^18^F]FDOPA PET acquisition these patients were antipsychotic naïve (no current or previous treatment) or free (not taking medication at scanning time with at least 6 months washout for oral medication or 6 months for depot medication). For this patient cohort, treatment response was defined as a total PANSS reduction of ≥ 50% at the initial follow-up, confirmed by remission criteria at six months [30]. FDOPA_02 includes twelve patients who met DSM-IV criteria for SCZ, treated with clozapine who had not responded to first-line antipsychotics and twelve patients who had responded to first-line antipsychotics [31]. All patients had received first-line antipsychotic drugs (including risperidone, olanzapine, and paliperidone) or clozapine for at least 12 weeks at the time of [^18^F]FDOPA PET acquisition. Finally, FDOPA_03 includes 24 patients who met DSM-IV criteria for SCZ [32]. In this dataset, non-responders were identified as patients with treatment-resistant SCZ who exhibited persistent symptoms (total PANSS score ≥ 75) despite receiving at least two sequential antipsychotic trials, each lasting a minimum of four weeks [32]. On the contrary, responders met treatment remission criteria with a scored ≤ 3 on all items of the PANSS questionnaire and had not experienced a symptomatic relapse in the 6 months prior to the study. All patients were taking antipsychotic medication at time of scanning other than clozapine. A summary of experimental variable and demographic information (age and sex) for each dataset is reported in Table 1.

**Table 1:** Demographic characteristics of patients (non-responders and responders)

	FDOPA_01				FDOPA_02				FDOPA_03			
	Non-responders (*N* = 13)		Responders (*N* = 12)		Non-responders (*N* = 12)		Responders (*N* = 12)		Non-responders (*N* = 12)		Responders (*N* = 12)	
	Mean	SD	Mean	SD	Mean	SD	Mean	SD	Mean	SD	Mean	SD
Age (years)	26.2	5.8	24.4	3.0	31.3	8.1	31.1	9.8	45.7	9.8	44.0	11.9
Radioactivity Injected (MBq)	147.4	16.2	144.6	6.3	366	25.9	363	33.3	180.0	5.5	183.6	4.3
Duration of illness (years)	2	2.9	1.0	1.7	12.1	6.5	9.3	9.0	16.1	8.6	16.2	10.1
Male/Female	10/3		12/1		9/3		8/2		5/7		6/6	
Scanner	Hi-Rez Biograph 6 (PET/CT)				Biograph 40 Truepoint (PET/CT)				ECAR HR + 962 (PET/CT)			
Imaging site	Invicro				South Korea				MRC Cyclotron Unit			

All [^18^F]FDOPA PET imaging data were acquired with a continuous dynamic acquisition (no arterial blood sampling), with simultaneous scanning and tracer injection, scanning lasting for 90–95 min. All participants received carbidopa (150 mg) and entacapone (400 mg) orally ~ 1 h before imaging to reduce the peripheral tracer metabolism. The [^18^F]FDOPA tracer was administered by intravenous bolus injection after the acquisition of brain CT or MRI for attenuation correction, depending on the scanner used at each imaging site. PET data reconstruction varied across imaging sites and scanner types, though all included correction for random noise, scatter, and tissue attenuation. More details on the acquisition and reconstruction parameters can be found in the original publication [28].

For [^18^F]FDOPA PET data quantification, the automated analysis pipeline proposed by Nordio et al. was applied consistently to all scans [28]. The pipeline quantifies K_i_^cer^ (unit 1/min), a kinetic parameter used as a proxy of dopamine synthesis capacity [34], and the Standardised Uptake Value ratio (SUVr), calculated as the ratio of the tracer activity in a target region to that in the reference region (i.e. mean cerebellum [^18^F]FDOPA PET activity). Specifically, SUVr was derived from a 15-min acquisition window starting 60 min after bolus administration. Further details on the analysis pipeline can be found in the original publication [28].

### Radiomic analysis

#### Delineation of volume of interest

**Fig. 1:**
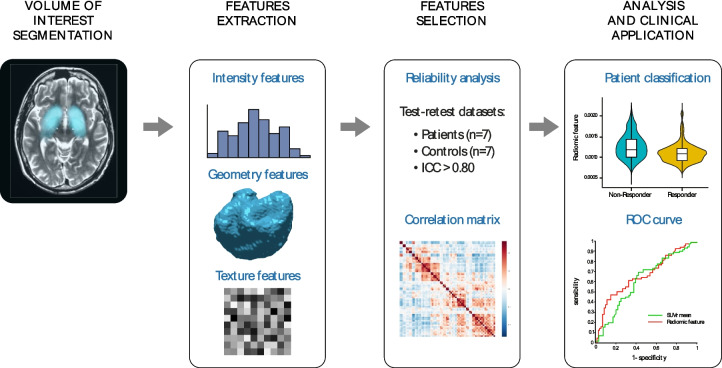
Schematic illustration of the analytical workflow used in this study. From left to right: VOI segmentation, feature extraction, feature selection, radiomic analysis, and clinical application

Figure 1 shows workflows of the radiomic analysis performed. The striatal region was considered as the volume of interest (VOI) for the radiomic analysis, since it is the main region involved in dopamine synthesis capacity. The striatal mask was derived from the Martinez atlas [35], previously co-registered to subject space, with two binary dilations on the striatal region [28]. K_i_^cer^ parametric maps were used to obtain the VOI mask by keeping voxels with a signal higher than 0.007 min^−1^. This threshold was chosen based on the quality assessment of a larger [^18^F]FDOPA dataset (*N* = 792, including healthy controls and patients with psychosis at different stages of the disease), for which K_i_^cer^ estimates smaller than this threshold were associated with poor image quality [28].

#### Feature extraction

Radiomic features were extracted using the Medical Image Radiomics Processor (MIRP) Python package [36]. MIRP comprises the following feature families: morphological, local intensity, intensity-based statistical, intensity histogram, intensity-volume histogram, grey level co-occurrence-based, grey level run length-based, grey level size zone-based, grey level distance zone-based, neighbourhood grey tone difference based, and neighbouring grey level dependence-based features.

MIRP package is Image Biomarker Standardization Initiative (IBSI) compliant [37], which is an independent international collaboration that aims to standardize the radiomic feature extraction in order to refine software agreement [37] and eventually improve the reproducibility of radiomic studies. The radiomic analysis was performed on the SUVr parametric maps since they have a higher signal-to-noise ratio than K_i_^cer^ parametric maps, allowing for a more robust analysis [38]. Since the data quantification pipeline already generated SUVr images with an isotropic voxel size of 2 mm, no additional resampling was required to harmonize voxel dimensions. Before the feature computation, individual striatal masks were further filtered by removing the voxels with an SUVr value lower than 1.50, which was estimated as a lower bound for the SUVr signal in healthy controls [28]. The images were then discretized with the fixed bin size method (biz size = 0.0125) [39], and features were computed with the 3D average aggregation method.

#### Reproducibility analysis and feature selection

Data from two independent datasets were used to conduct a reproducibility analysis. The first dataset comprised [^18^F]FDOPA PET test–retest imaging data from 7 healthy controls [40], while the second dataset comprised [^18^F]FDOPA PET test–retest imaging data from 7 patients with SCZ, scanned twice, before and after taking placebo medication for approximately one month.

The Intraclass Correlation Coefficient (ICC) [41] was computed separately for the healthy controls and patients datasets, and only repeatable radiomic features with an ICC > 0.80 for both cases were retained for further analyses. This threshold, which is consistent with a biomarker of good reliability [42], was selected based on the ICC performances obtained from [^18^F]FDOPA dynamic PET analysis, combining both healthy controls and patients with SCZ [28]. With this approach we made sure to select radiomic features for striatal [^18^F]FDOPA PET imaging as reliable K_i_^cer^ estimates derived from dynamic PET imaging analysis.

Radiomic features are known to produce redundant information and to generally be highly correlated [43]. Dimensionality was reduced through hierarchical clustering based on the Spearman correlation coefficient (ρ) on the whole test–retest datasets.

The absolute correlation was computed for each pair of features and then used to create a dissimilarity matrix. Hierarchical clustering was then applied, to obtain clusters where each pair of features had a ρ of at least 0.90. Eventually, for each cluster, the feature with the highest ICC was selected, combining data for healthy controls and patients.

#### Feature harmonisation

Harmonisation to correct for scanner effects and site inconsistencies was applied directly to the selected features using the NeuroCombat method [44, 45] (NeuroCombat python library version 0.2.10 +). We chose this approach for feature harmonization because of its broad applicability, straightforward statistical implementation, and proven effectiveness [46].

Harmonization was performed for both controls and patients using the same reference scanner. The effects of age and sex as covariates were preserved.

### Statistical analysis

Statistical analysis was performed using SPSS (version 29). The effect of sex and age was evaluated separately for healthy controls and patients, using a χ^2^ test for the categorical covariate (sex) and the Wilcoxon test for the continuous variable (age). An ANOVA was performed to evaluate the influence of age and sex on radiomic features, for healthy controls and patients.

Statistical differences between features in patients who were *responders* and *non-responders* were evaluated with a two-way ANOVA, with sex and age effects in the model and corrected with a False Discovery Rate (FDR) correction. Due to the high heterogeneity of the patient data at different illness stages, a separate ANOVA was performed for each patient subgroup. The mean striatal SUVr was included in the analysis, as the reference standard quantification metric. Statistical differences between the radiomic features in healthy controls vs. responders and healthy controls vs. non-responders were also evaluated with a two-way ANOVA, with sex and age effects included in the model. For the response classification, the Receiver Operating Characteristics (ROC) area under the curve (AUC) was computed using the pROC [47] package in R (version 4.2.1). The AUC was calculated for all the features and used as the performance index of the feature to identify non-responders within the whole cohort of patients and for each patient dataset. The AUCs were then compared between groups using the default options of the pROC [47] package in R (version 4.2.1). To validate the classification a random forest with k-fold cross validation (k = 10) was performed in R (version 4.3.1) using the packages randomForest (version 4.7.1.1) and caret (version 6.0.94) [48].

## Results

### Radiomic analysis

#### Feature extraction, selection, and harmonisation

With the test–retest datasets, a total of 146 out of 177 features survived the reproducibility criteria (ICC > 0.80) in the patient group, while 85 met criteria within the healthy control group. Combining the two analyses, 81 features met reproducibility criteria in both groups (~ 45% of the initial set of features).

Unsupervised cluster analysis based on Spearman correlation coefficient resulted in 15 clusters, and from each one of them, the feature with the highest ICC was selected as representative. Table 2 reports all the features selected along with their physiological meaning and the corresponding ICC for healthy controls and patients (ICC: 0.81–0.99).

**Table 2:** Reproducibility analysis

Feature family	Feature	Meaning	Controls ICC	Patients ICC
Morphological	Elongation	Ratio of the major and minor principal axis lengths (eccentricity of the ROI)	0.895	0.930
	Maximum 3D diameter	Distance between the two most distant vertices	0.892	0.850
	Flatness	Ratio of the major and least axis lengths (flatness of the volume relative to its length)	0.898	0.885
	Major axis	Length of the major axis associated with the highest eigenvalue	0.991	0.963
Intensity-based statistical	Intensity-based quartile coefficient of dispersion	Measure of the dispersion of grey levels	0.929	0.907
	Median absolute deviation	Measure of the dispersion from the sample median	0.928	0.932
Intensity histogram	Median	Median value of the histogram	0.902	0.933
Grey level co-occurrence matrix	Joint maximum	Most common grey level co-occurence	0.817	0.835
	Contrast	Measure of grey level variations	0.875	0.926
	Energy	Energy of the probability distribution of grey level co-occurences	0.920	0.931
Grey level run length matrix	Run length non uniformity	Distribution of runs over the run lengths	0.810	0.858
Grey level size zone matrix	Zone size entropy	Quantity of information contained in a matrix where the element s_*ij*_ = the number of zones with grey level *i* and size *j*	0.927	0.941
Grey level distance zone matrix	Zone distance non uniformity	Measure of the distribution of the number of zones over the different distances	0.842	0.843
Neighbourhood grey tone difference matrix	Complexity	Quantification of the non-uniformity of the texture and rapid changes in grey levels	0.848	0.928
Neighbouring grey level dependence matrix	Low dependence low grey level emphasis	Emphasis on the number of groups with a low number of voxels centered on a low grey level voxel	0.862	0.962

The batch effect on the selected features was then removed, using the NeuroComBat harmonization method, as shown in Supplementary Fig. 1 for the healthy control group. Supplementary Table 1 shows the association between the radiomic features and the imaging sites, which is completely removed after harmonization.

### Relationship between radiomic features and demographic variables

The association between striatal [^18^F]FDOPA PET radiomic features and subject demographics (age and sex) for healthy controls and patients is reported in Table 3. We found different associations between features and demographics in controls and patients. Within the control group, all morphological features were significantly associated with sex, while contrast and run length non uniformity were associated with age. Within the patient group, all features were significantly associated with sex, excluding the elongation and the flatness. On the other hand, only few features were significantly associated with age (elongation, flatness, contrast, run length non uniformity, zone distance non uniformity, complexity).

**Table 3:** Association with sex and age

		Controls			Patients		
Feature family	Feature	F	Gender	Age	F	Gender	Age
Morphological features	Elongation	6.07	0.015		25.04		< 0.001
	Maximum 3D diameter	31.44	< 0.001		25.04	< 0.001	
	Flatness	21.21	< 0.001		7.15		0.008
	Major axis	39.62	< 0.001		23.77	< 0.001	
Intensity-based statistical features	Intensity-based quartile coefficient of dispersion				4.08	0.046	
	Median absolute deviation				12.32	< 0.001	
Intensity histogram	Median				17.73	< 0.001	
Grey level co-occurrence matrix	Joint maximum				5.66	0.019	
	Contrast	5.85		0.017	19.56	< 0.001	0.002
	Energy				7.24	0.008	
Grey level run length matrix	Run length non uniformity	4.18		0.043	9.71	0.007	0.006
Grey level size zone matrix	Zone size entropy				13.75	< 0.001	
Grey level distance zone matrix	Zone distance non uniformity				20.37	< 0.001	0.002
Neighbourhood grey tone difference matrix	Complexity				21.25	< 0.001	0.024
Neighbouring grey level dependence matrix	Low dependence low grey level emphasis				12.65	< 0.001	

Results from ANOVA, using AGE and GENDER as regressors. Significant F-statistics and *P*-values for age and gender separately are displayed in the table after FDR correction

#### Comparison and classification of treatment response

The two patient groups, *responders* (39 males and 32 females, age 31.5 ± 11.2 years) and *non-responders* (39 males and 25 females, age 32.6 ± 10.5 years), were matched for both sex (*p* = 0.480) and age (*p* = 0.281).

Table 4 shows adjusted *p*-values of radiomic features significantly different between responders and non-responders, after FDR correction for multiple comparisons. Given the observed heterogeneity in illness stage between patient subgroups, ANOVA analysis was conducted separately within each patient subgroup (i.e. FDOPA_01, FDOPA_02, FDOPA_03). In each dataset, responders and non-responders were matched for both sex and age. All features differed significantly between responders and non-responders in at least one dataset, excluding the maximum 3D diameter of the main region of interest (i.e. striatum).

**Table 4:** Analysis of treatment response

Feature family	Feature	All patients	FDOPA_01	FDOPA_02	FDOPA_03
Morphological	Elongation	0.031			0.007
	Maximum 3D diameter				
	Flatness	0.040			< 0.001
	Major axis				0.017
Intensity-based statistical	Intensity-based quartile coefficient of dispersion	0.001	< 0.001	0.003	0.006
	Median absolute deviation	0.006	< 0.001	0.003	0.004
Intensity histogram	Median	0.011		0.004	0.004
Grey level co-occurrence matrix	Joint maximum	< 0.001	< 0.001	0.002	< 0.001
	Contrast	0.031	0.003	0.004	0.015
	Energy	0.005	< 0.001	0.010	0.001
Grey level run length matrix	Run length non uniformity	0.015	< 0.001	0.038	0.004
Grey level size zone matrix	Zone size entropy	0.011	0.005	0.007	0.001
Grey level distance zone matrix	Zone distance non uniformity	0.007	< 0.001	0.013	0.032
Neighbourhood grey tone difference matrix	Complexity		0.004	0.010	0.011
Neighbouring grey level dependence matrix	Low dependence low grey level emphasis	0.040		0.021	0.005
Mean SUVr		0.005	0.003	0.012	< 0.001

Results from One-way ANOVA, using AGE and GENDER as covariates. Significant *P*-values from group comparison (responders vs non-responders) are displayed in the table after FDR correction

**Fig. 2:**
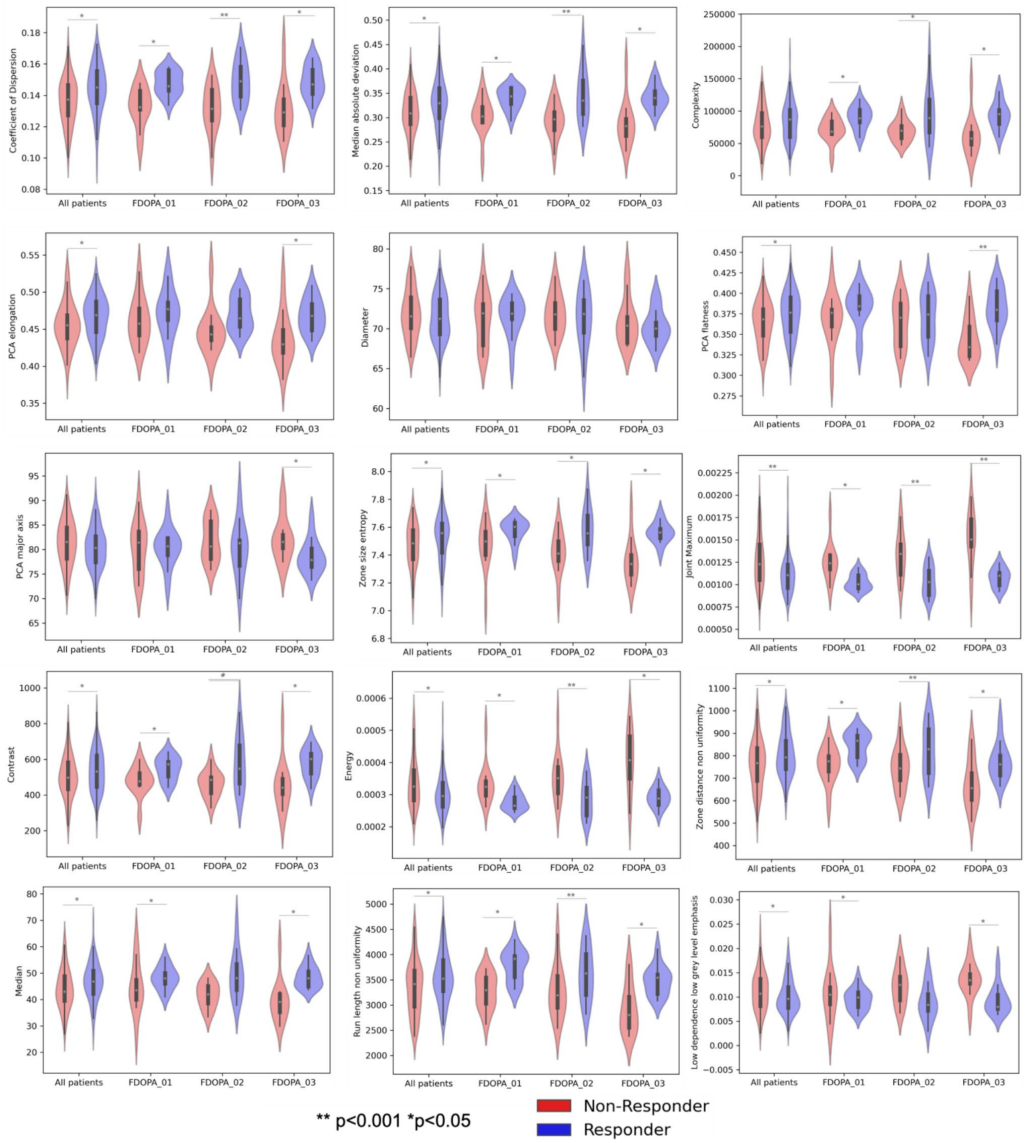
Distribution of radiomic features in all patients and in each dataset (FDOPA_01, FODPA_02, FDOPA_03) for non-responders (red) and responders (blue)

When examining the feature distribution between responders and non-responders (Fig. 2), higher values were obtained for responders compared to non-responders, excluding the grey level co-occurrence matrix (GLCM) joint maximum and energy. In addition, most features differed significantly between responders and healthy controls (Table 5). Three features (coefficient of dispersion, GLCM joint maximum, run length uniformity) differed significantly between healthy controls and both responders and non-responder groups, though in the opposite directions.

**Table 5:** Feature differences between non-responders and responders versus healthy controls

		Non-Responders vs Controls		Responders vs Controls	
Feature family	Feature	F	Mean difference	F	Mean difference
Morphological	Elongation			5.99*	0.013
	Maximum 3D diameter				
	Flatness			5.21*	0.010
	Major axis				
Intensity-based statistical	Coefficient of dispersion	4.28*	−0.007	8.06*	0.009
	Median absolute deviation			7.77*	0.270
Intensity histogram	Median			6.82*	4.33
Grey level co-occurrence matrix	Joint maximum	5.13*	1.3E-04	10.7*	−1.7E-04
	Contrast	5.10*	−61.2		
	Energy			8.44*	−4.6E-05
Grey level run length matrix	Run length non uniformity	5.02*	−224	5.57*	224
Grey level size zone matrix	Zone size entropy			10.5*	0.114
Grey level distance zone matrix	Zone distance non uniformity	4.59*	−44.3		
Neighbourhood grey tone difference matrix	Complexity				
Neighbouring grey level dependence matrix	Low dependence low grey level emphasis			5.17*	−0.002
Mean SUVr				12.6**	0.135

Results from One-way ANOVA, using AGE and GENDER as covariates. Significant F-statistics and mean difference from group comparison (responders vs controls, non-responders vs controls) are displayed in the table after FDR correction

*indicates *p*-value < 0.05; ** indicates *p*-value < 0.001

The classification performances between responders and non-responders expressed with the AUC ROC are reported in Table 6. When considering the entire patient dataset, the feature with the highest AUC value was the GLCM joint maximum (AUC = 0.66). The GLCM joint maximum feature was also the feature with highest AUC for the subgroups FDOPA_01 (AUC = 0.88) and FDOPA_02 (AUC = 0.85), while for FDOPA_03 the highest AUC was observed for the median value (AUC = 0.92).

**Table 6:** Classification of patients treatment response with ROC analysis

Feature family	Feature	AUC ROC All patients	AUC ROC FDOPA_01	AUC ROC FDOPA_02	AUC ROC FDOPA_03
Morphological	Elongation	0.62^*^	0.61^NS^	0.79^*^	0.82^*^
	Maximum 3D diameter	0.55	0.46	0.47	0.58
	Flatness	0.60^*^	0.71^NS^	0.58^NS^	0.87^*^
	Major axis	0.57^NS^	0.56^NS^	0.44^NS^	0.76^*^
Intensity-based statistical	Coefficient of dispersion	0.66^*^	0.86*	0.81*	0.85^*^
	Median absolute deviation	0.63^*^	0.82*	0.77*	0.89^*^
Intensity histogram	Median	0.63^*^	0.69^NS^	0.77*	0.92^*^
Grey level co-occurrence matrix	Joint maximum	0.66^*^	0.88*	0.85*	0.87^*^
	Contrast	0.58^NS^	0.74*	0.72^NS^	0.85^*^
	Energy	0.62^*^	0.86*	0.78*	0.86^*^
Grey level run length matrix	Run length non uniformity	0.61^*^	0.85*	0.70^NS^	0.82^*^
Grey level size zone matrix	Zone size entropy	0.62^*^	0.73*	0.75*	0.89^*^
Grey level distance zone matrix	Zone distance non uniformity	0.60^*^	0.82*	0.70^NS^	0.78^*^
Neighbourhood grey tone difference matrix	Complexity	0.56^NS^	0.76*	0.69^NS^	0.86^*^
Neighbouring grey level dependence matrix	Low dependence low grey level emphasis	0.60^*^	0.60^NS^	0.73*	0.83^*^
Mean SUVr		0.62^*^	0.79*	0.77*	0.90^*^

The highest AUC ROC values for each subgroup are indicated in bold

*NS* non significant

*indicates *p*-value < 0.05

Compared to these performances, the striatal mean SUVr (reference standard for [^18^F]FDOPA PET analysis) had a lower AUC in all the groups (all the patients AUC = 0.62, FDOPA_01 AUC = 0.79, FDOPA_02 AUC = 0.77, FDOPA_03 AUC = 0.90), although the differences did not reach statistical significance as measured with the bootstrap test implemented in the pROC package (FDOPA_01: D = −1.249, *p* = 0.211; FDOPA_02: D = −1.052, *p* = 0.293; FDOPA_03: D = −0.809, *p* = 0.418). The AUC results were confirmed by a multivariate random forest classification in a tenfold cross-validation, when all the features were combined in a model to classify responders vs non responders and only GLCM joint maximum was chosen reaching the highest classification accuracy (mean decrease accuracy = 10.55 and mean decreased Gini = 7.66).

## Discussion

In this study, radiomics was applied to [^18^F]FDOPA brain PET imaging data from healthy participants and patients with psychosis at different illness stages. Fifteen features were identified as the most reliable and selected as the optimal representation of the radiomic feature set (*n* = 177). Excluding the maximum 3D diameter, all other features were able to distinguish treatment responders from treatment non-responders confirming that striatal [^18^F]FDOPA PET imaging captures information of treatment response in SCZ and that this information is available beyond dynamic acquisition and full compartmental modelling for quantification. Among the radiomic features the GLCM joint maximum, which weights the most prevalent pair of neighbouring intensity values, gave the highest discrimination accuracy. This was also seen in comparison to the reference standard of striatal mean SUVr. Together, these findings confirm [^18^F]FDOPA PET as quantitative imaging method to support classification of treatment response in psychotic disorders.

### Radiomics for classification of treatment response in psychosis

In this study, fourteen out of the fifteen test–retest repeatable radiomic features differed significantly between responders and non-responders. SCZ is characterized by high heterogeneity in biological and clinical characteristics [49] and antipsychotic treatment interferes with striatal dopamine function [50]. Together, these two might cause the observed variation among the signal measured in the patient cohorts. Nevertheless, these results were consistent when homogenous subgroups of patients, including antipsychotic naïve/free patients, were analysed independently. Our results indicate that differences between responders and non-responders are related to the texture features rather than structural/morphological properties of the volume of interest. Morphological features were less significantly different between responders and non-responders, with the maximum 3D diameter non-significant in any subgroups of patients. The elongation of the region of interest was higher in the responders’ group, which could be explained by the increased dopaminergic activity in the striatal region.

Features related to texture inhomogeneity were higher for patients who responded to antipsychotics compared to non-responders. The higher values of these inhomogeneity features within the responders’ group is consistent with increased dopamine synthesis capacity that typically characterised this class of patients. Preliminary analysis from our group using covariance-based statistics applied to [^18^F]FDOPA PET confirmed these results, showing a higher degree of signal perturbation in striatal regions in responders compared to non-responders [51].

The non-responders had higher values only for GLCM joint maximum and the GLCM energy features. Features in this family were not computed on the SUVr map itself, rather on a matrix that represented the probability that two adjacent voxels would have specific discretized intensity values. Such probability matrix is used to compute the energy of the probability distribution (GLCM energy) and extract the probability of the most common co-occurrence pair. These features had higher values within the non-responders group, which implies a more regular and homogeneous pattern in the distribution of the signal in the striatal region compared to responders. Although it is difficult to derive a clean biological interpretation of these metrics in the context of dopamine synthesis, the superior discrimination accuracy of the GLCM joint maximum might be exploited for clinical translation of [^18^F]FDOPA PET imaging in SCZ.

Interestingly, radiomics allowed us to identify several statistical differences between responders and non-responders, but without outperforming the reference mean SUVr. This finding was unexpected and might be due to the limited sample size of the study. Further analyses using independent samples are needed to clarify this association. Nonetheless, within the responders’ group, most of the radiomic features were significantly different from the healthy controls. This ‘deviation’ from normality further supports altered dopamine synthesis capacity in the striatal region in SCZ and in particular in patients who respond to antipsychotic treatment, as indicated by the significant difference of the mean SUVr signal [11–13, 15, 52], compared to non-responders.

### Age and sex effect

A clear effect of age and sex was found in the patients’ cohorts, which was less evident within healthy controls. In controls, an association with age was observed for two features (contrast and run length non-uniformity). This result requires further investigation due to the relatively limited age range of the controls (age 28.5 ± 7.9 years). Sex was related only to the morphological features. This could be explained by the anatomical properties of the area, as the striatum tends to be smaller in women [53].

These associations differed in the patient cohorts, where a larger group of features was found to be associated with both sex and age. Some evidence suggests that male and female patients with SCZ differ in cognitive functions and neuroanatomy, though findings are inconsistent [54, 55]. In addition, the age of onset of psychotic symptoms and the prevalence of the disorder varies between men and women [56]. Age appears to be related to cognitive changes in SCZ patients in a different way than those observed in a healthy population [57]. In addition, patients with SCZ show accelerated brain ageing and increased risk of dementia in later life [58, 59].

Among morphological features in the patients’ group, two features (elongation and flatness) showed significant age-related differences, while the other two (Major axis and maximum 3D diameter) showed sex-related differences. While there are contrasting opinions regarding the presence of a volume difference in patients compared to controls [60, 61], changes in shape do exist [56] and may also be influenced by medication [62].

### Radiomics and striatal [^18^F]FDOPA PET imaging

A major limitation of radiomics is the reproducibility of findings, as computation of features is heavily dependent on imaging modality, reconstruction protocol and scanner type. discretization method, and the VOI segmentation [63].

In this study, we used data acquired from different PET scanners and research centres. To account for this variability, we applied NeuroComBat for feature harmonization to mitigate scanner-derived biases that may persist despite image normalization (i.e., isotropic voxel resampling and intensity normalization to the reference region). We acknowledge that emerging deep learning-based methods, such as CNNs and CycleGANs [64, 65], offer promising alternatives for image harmonization. While these approaches aim to harmonize images at the voxel level, their application to PET imaging remains an open area of research and future studies are needed to assess their impact on radiomic feature stability.

In oncology, radiomic studies typically use gross tumour volume as the VOI. Here, radiomic features were extracted from the striatum, since it is the main region of interest when studying dopamine synthesis in the brain. The segmentation of the striatum was computed on the [^18^F]FDOPA PET signal of the K_i_^cer^ parametric map, since analysis over a larger number of subjects was available, which allowed determination of a threshold for the VOI segmentation (0.007 1/min), ensuring signal in the voxels was not due to motion artifacts [28]. The striatal mask was then used on the SUVr parametric map of each subject to extract radiomic features. While dopamine synthesis is concentrated in the striatal area, future radiomic studies could be extended to other regions of interest in the brain, especially in those with relevant [^18^F]FDOPA PET signals (e.g., substantia nigra and pallidum [28]). Similarly, future studies should consider the potential effect of brain lateralisation of dopamine function [66] which was not considered in this application. Nevertheless, our reproducibility analysis on test–retest imaging data to select features with the highest reproducibility, demonstrated that radiomic analysis can achieve consistent test–retest performance to [^18^F]FDOPA PET imaging dynamic analysis.

## Conclusions

This study demonstrates that radiomics applied to [^18^F]FDOPA PET imaging data can capture and quantify brain alterations in dopamine synthesis capacity in patients with psychotic disorders and discriminate antipsychotic treatment responses. Although offering modest classification improvements over standard imaging, it confirms both the alteration of [^18^F]FDOPA PET striatal signal in psychosis and [^18^F]FDOPA PET ability to differentiate treatment response, even when neuroimaging data are acquired with statistic protocol. Despite these promising results, multi-site large-scale prospective studies would be necessary to confirms [^18^F]FDOPA PET ability as a clinically viable biomarker to guide treatment choice from an early stage of psychosis.

## Supplementary Information

Below is the link to the electronic supplementary material.

Supplementary file1 (DOCX 3223 KB)

## Data Availability

The datasets generated during and/or analysed during the current study are available from the corresponding author on reasonable request.
