# Supplementary material for: Radiomic Analysis of Striatal [18F]FDOPA PET Imaging in Patients with Psychosis for the Identification of Antipsychotic Response
Source: Mol Imaging Biol. 2025 May 5;27(3):365–78. doi: 10.1007/s11307-025-02014-3 (PMC12162767; doi:10.1007/s11307-025-02014-3)
Supplement: Supplementary file 1 — Supplementary file1 (DOCX 3223 KB) [file 11307_2025_2014_MOESM1_ESM.docx]

**Radiomic analysis of striatal [^18^F]FDOPA PET imaging in patients with psychosis for the identification of antipsychotic response - SUPPLEMENTARY**

**Supplementary Figures**

Supplementary Figure 1: Effect of ComBat harmonization on all the 15 selected features for the healthy controls group.


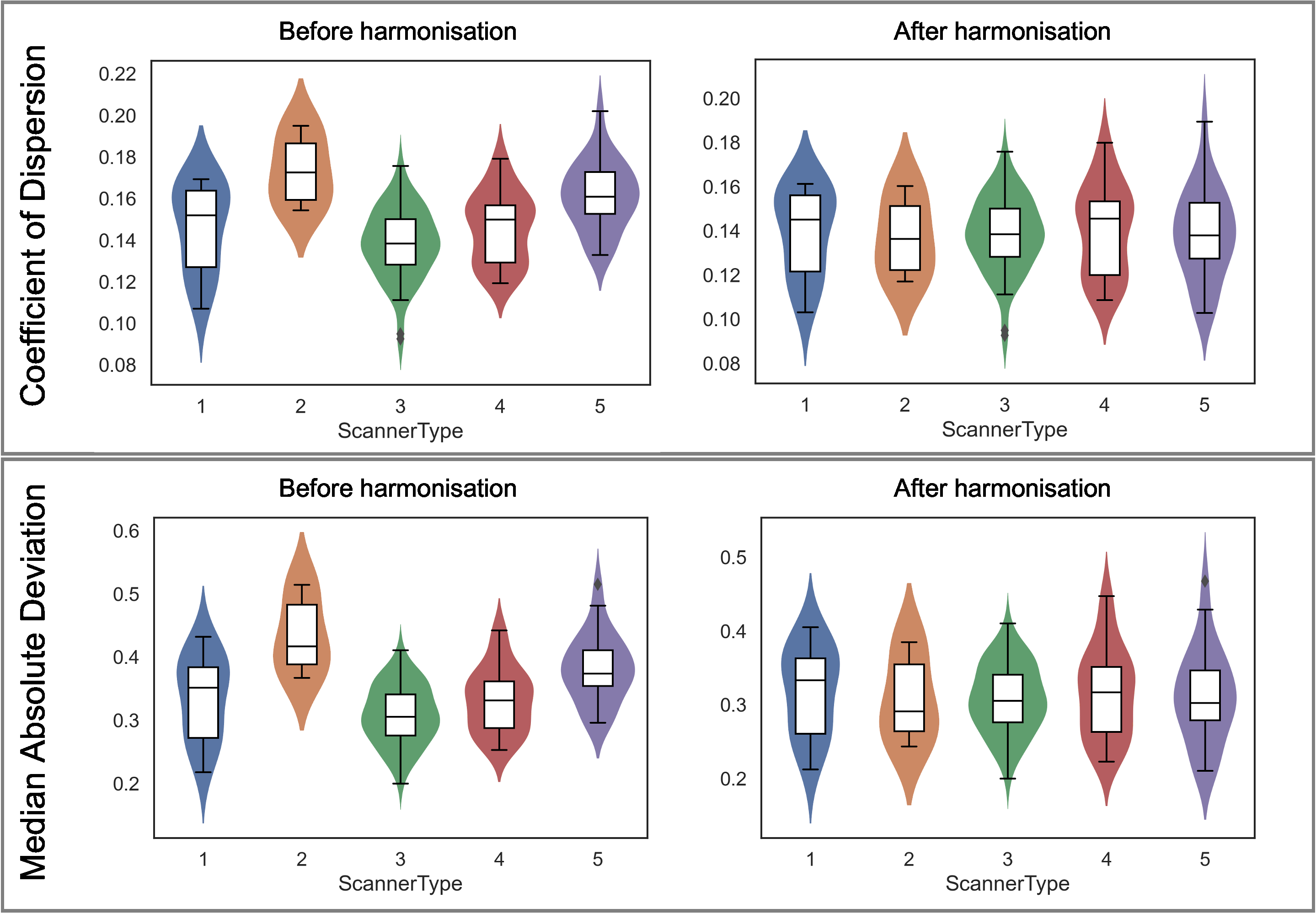

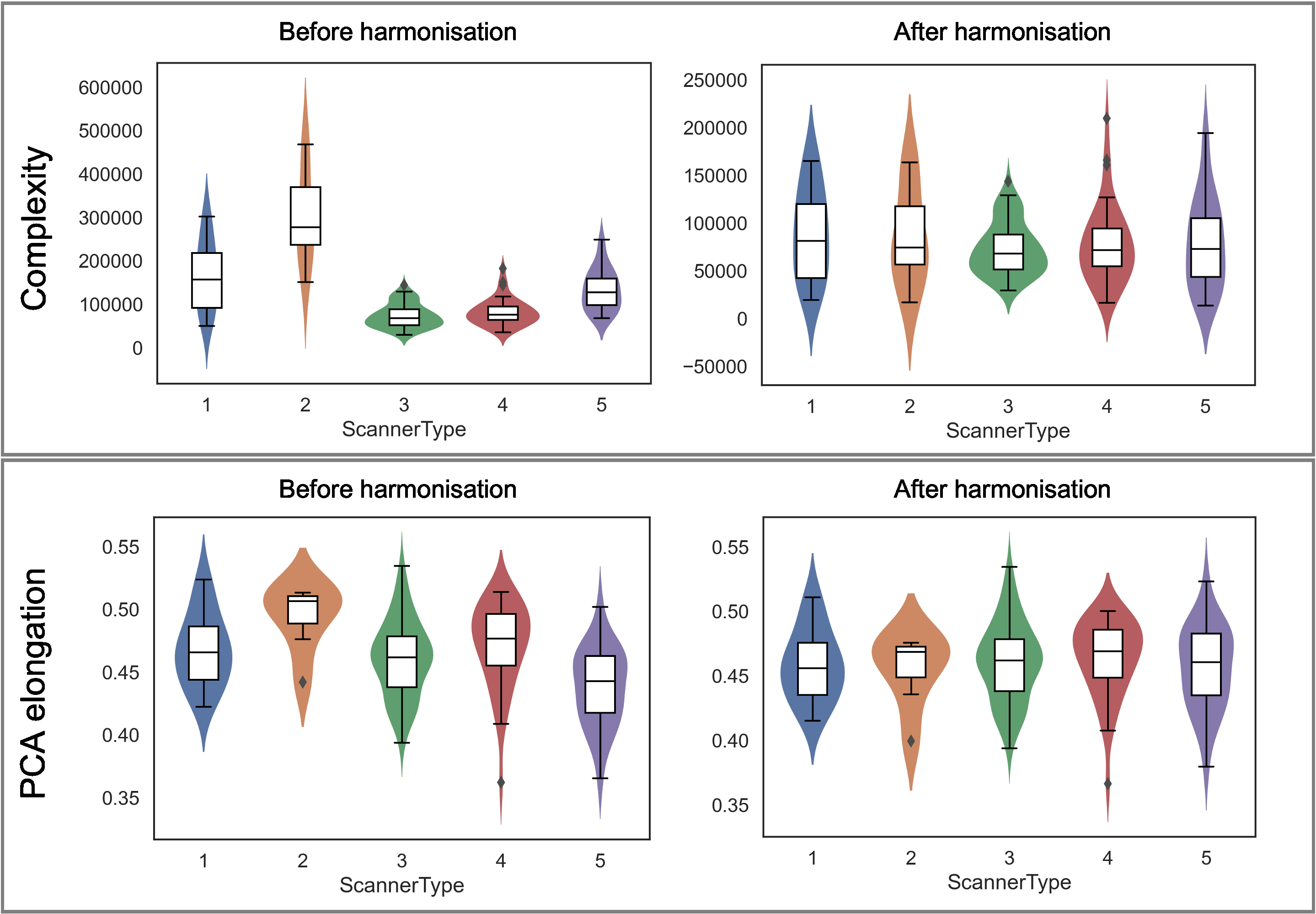


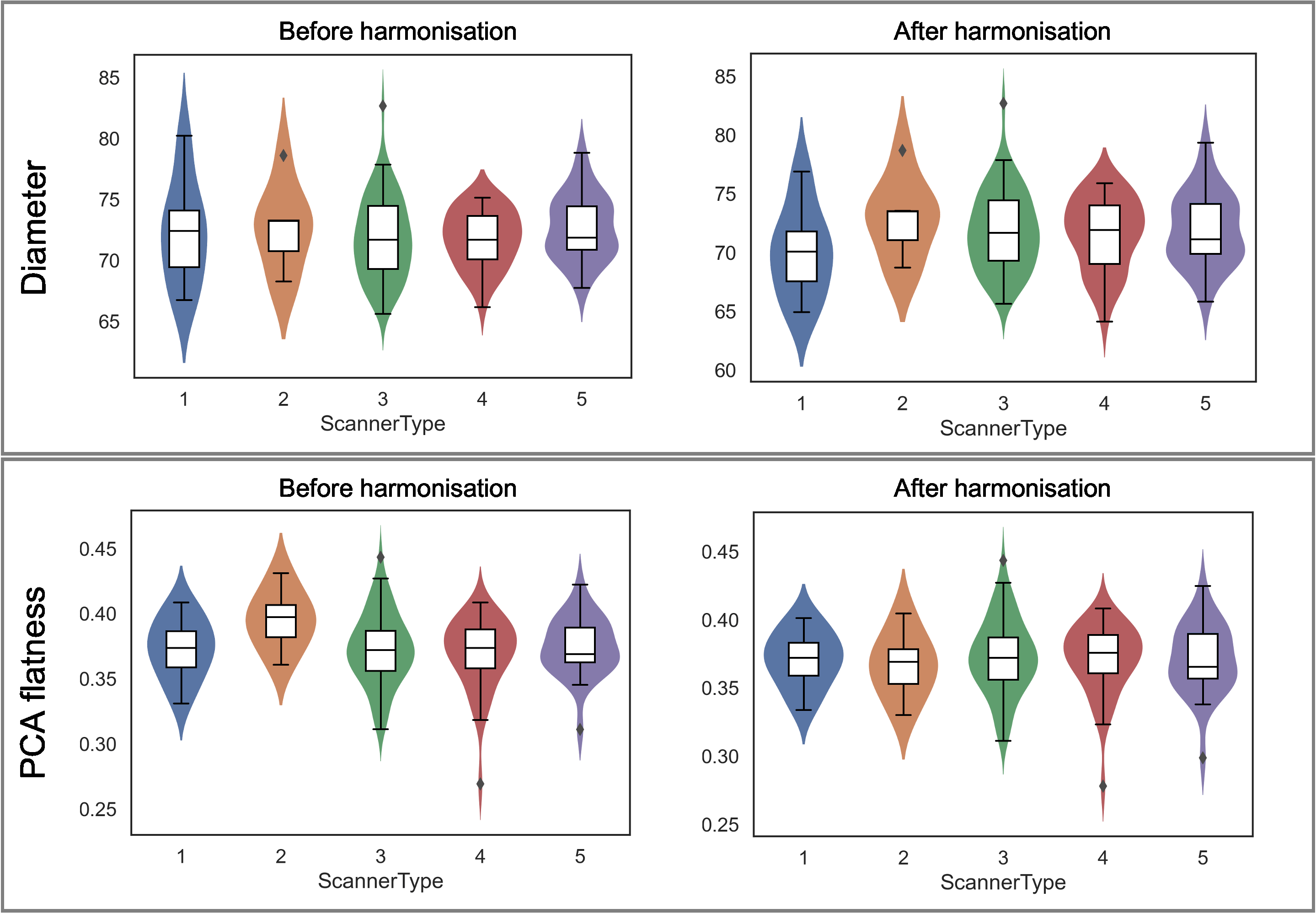

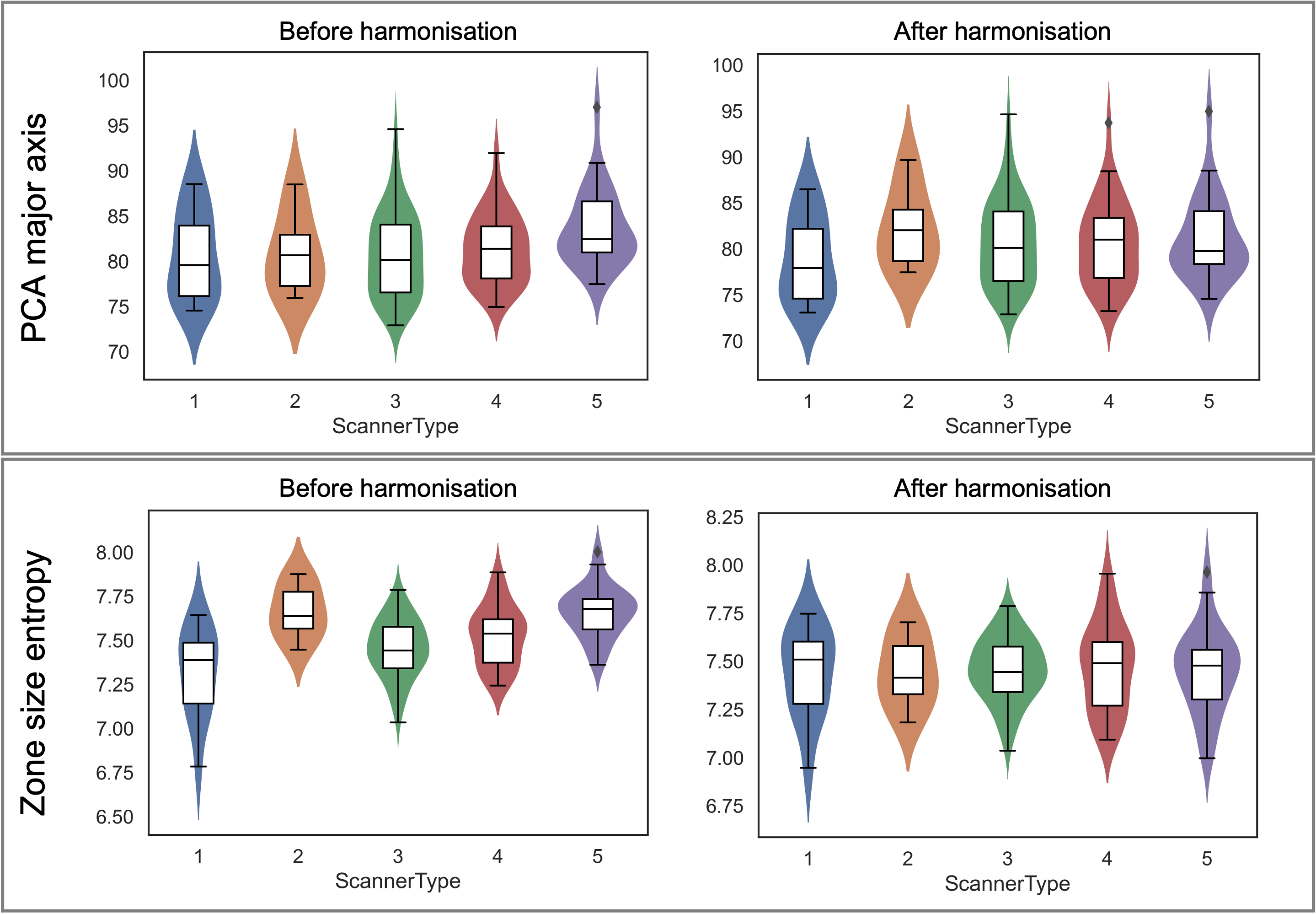


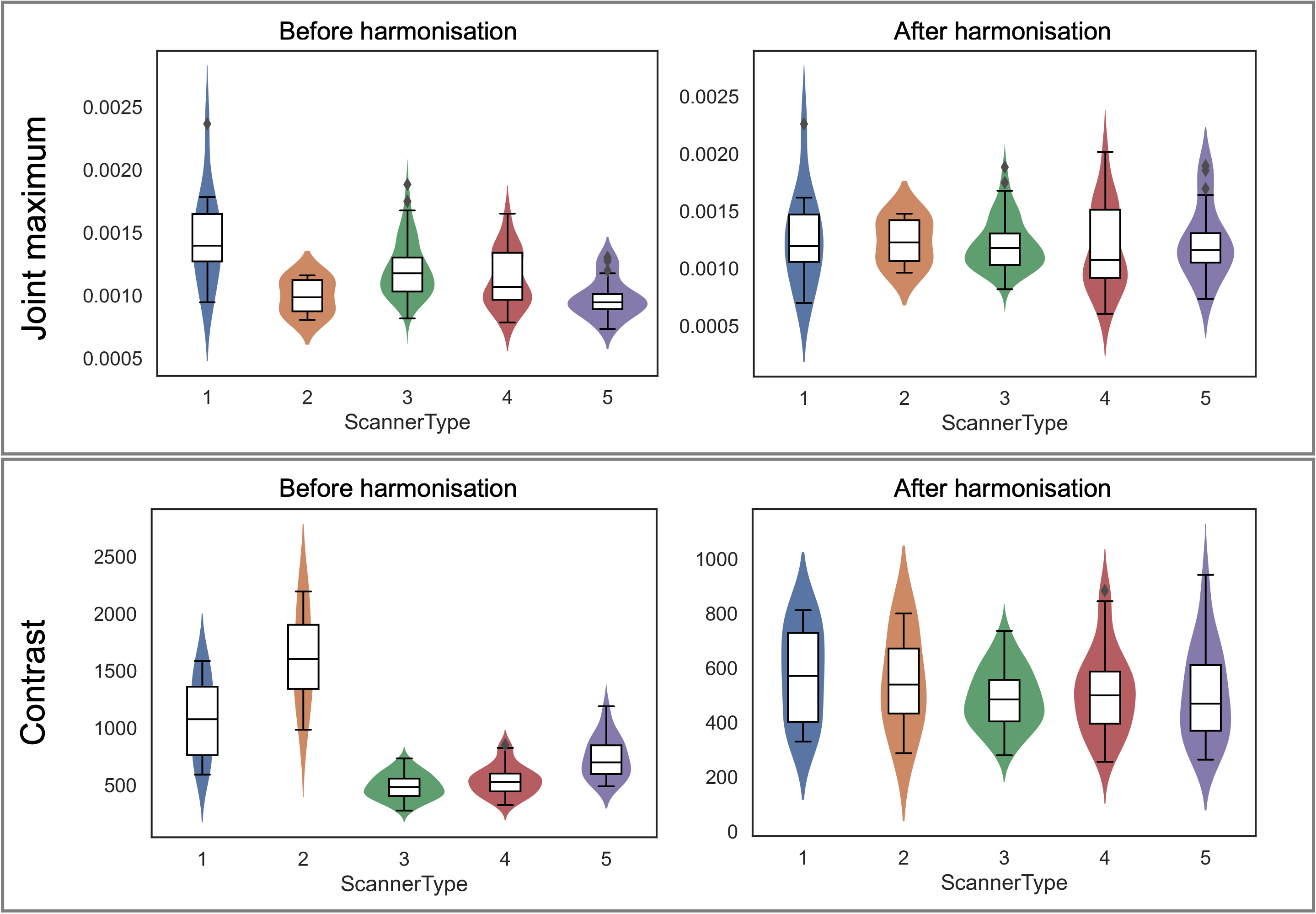

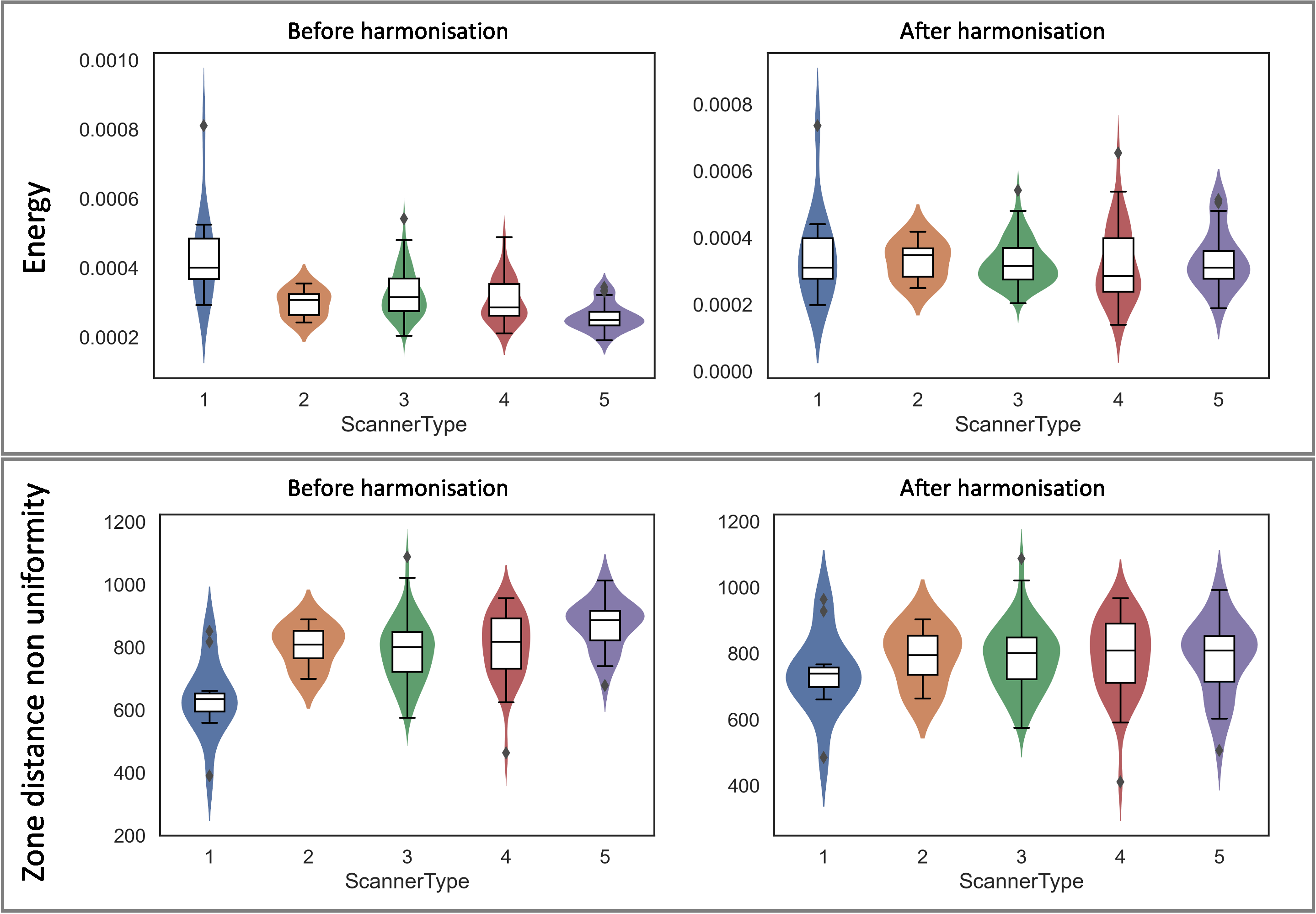


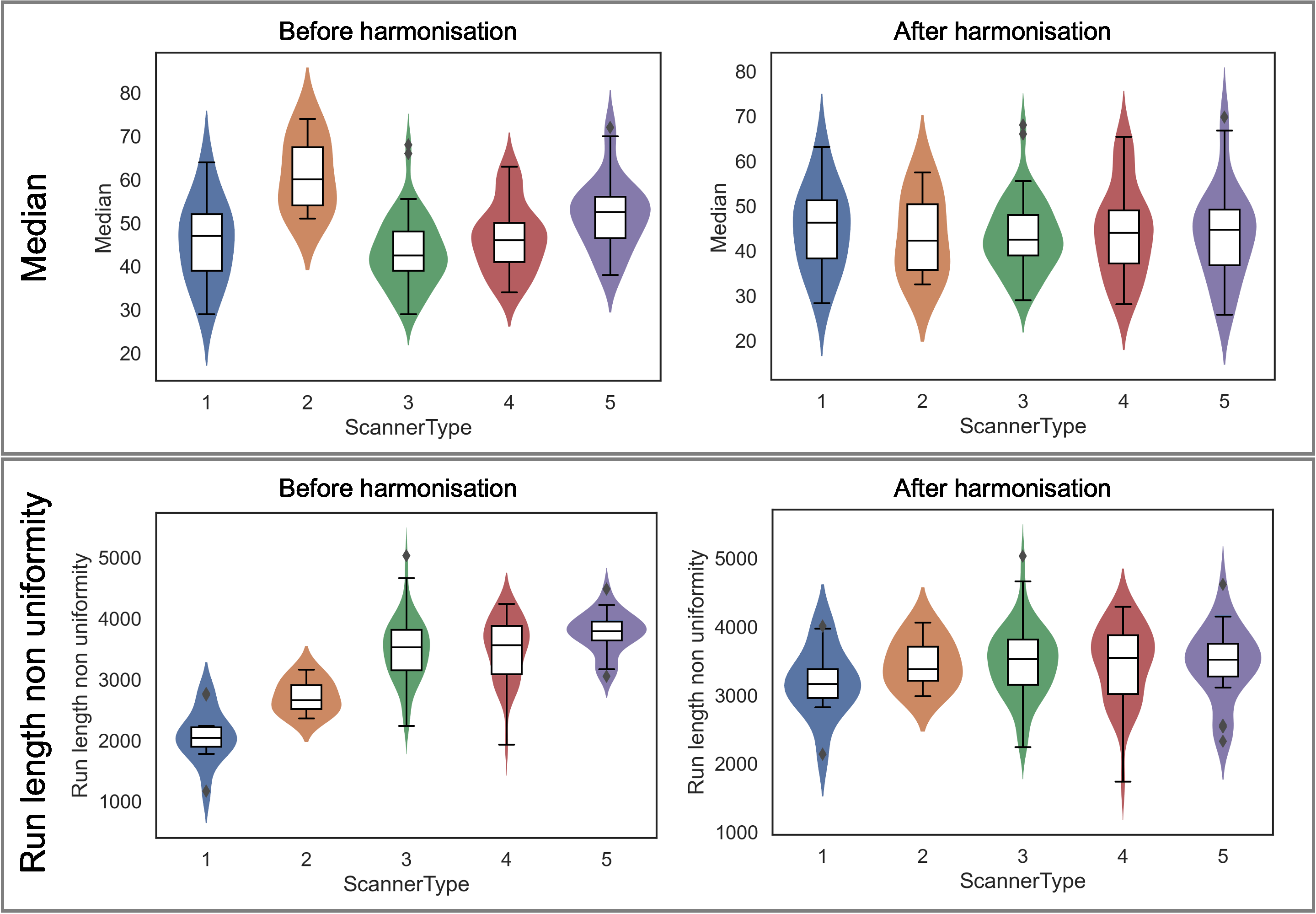

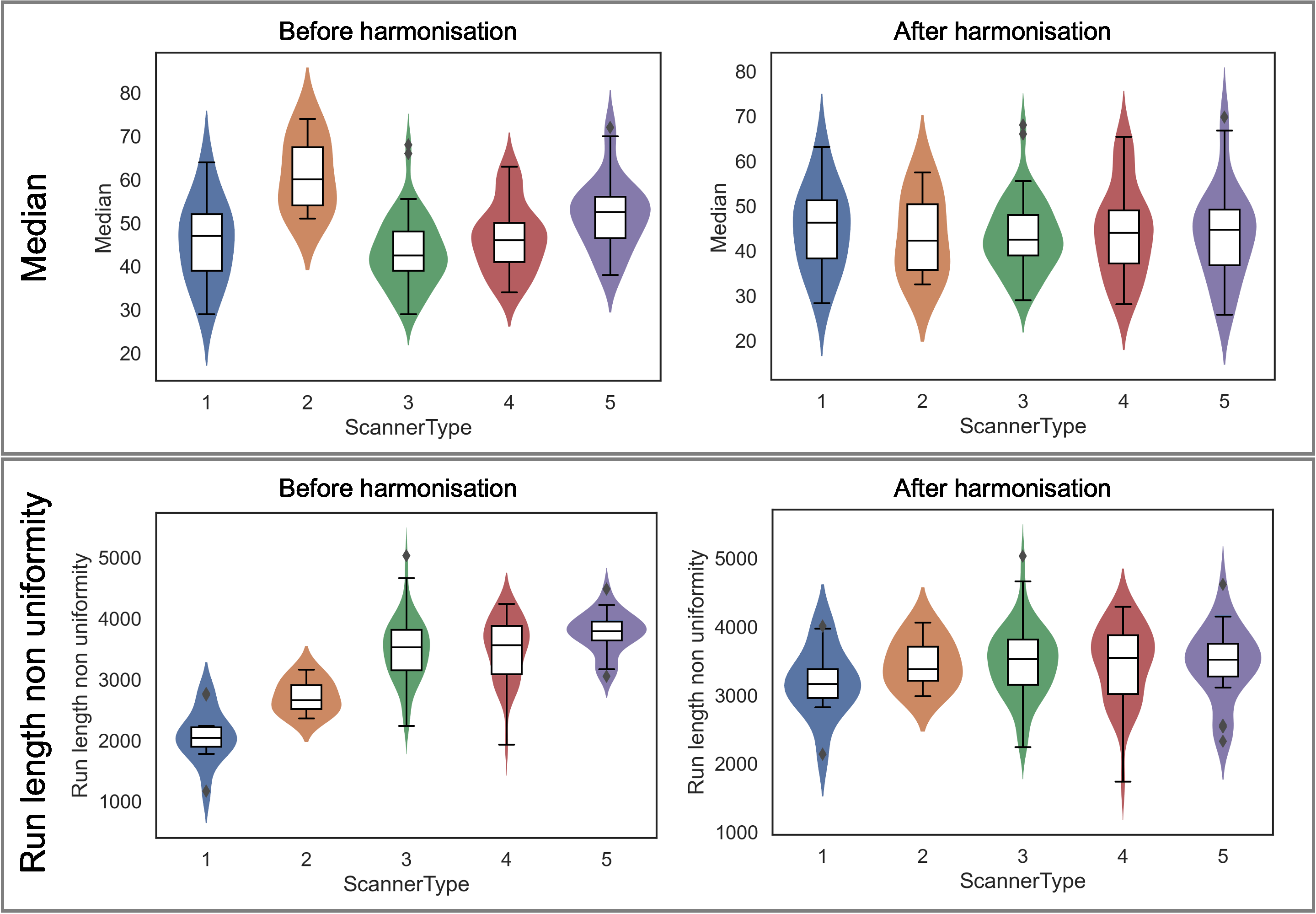


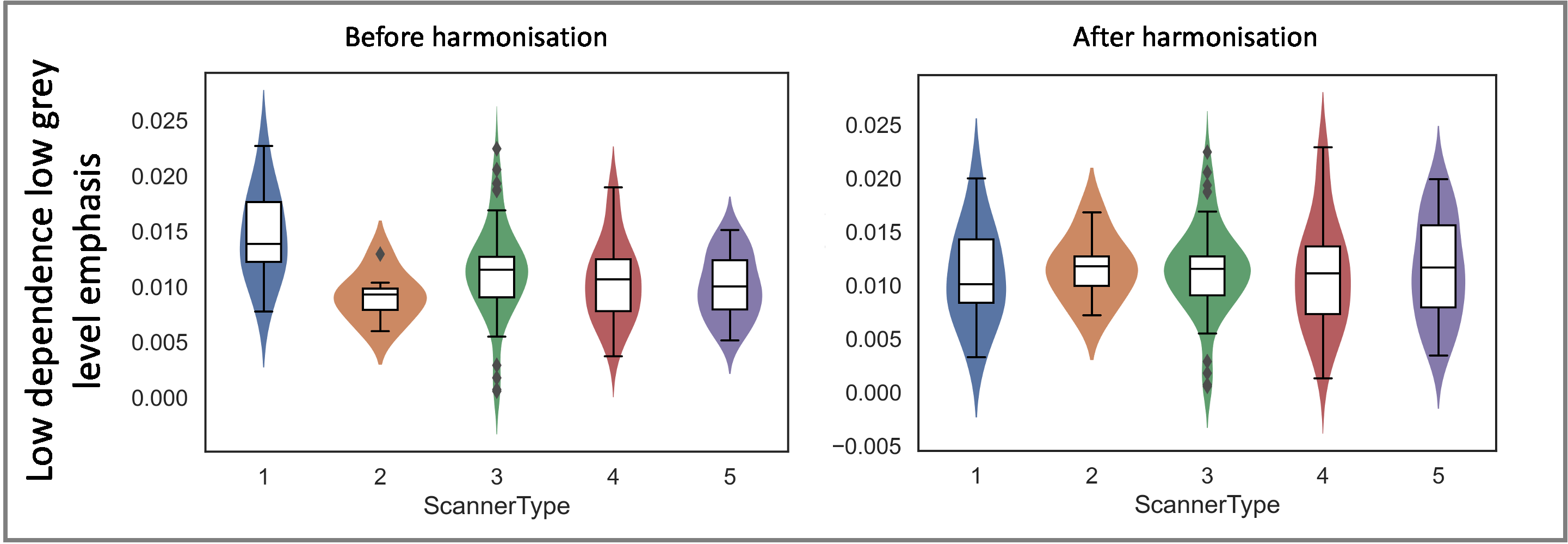


**Supplementary Tables**

Supplementary Table 1: ANOVA analysis to test association between radiomic features with the imaging site, before and after ComBat harmonization. Significant P-values are reported after FDR correction.

|  |  | **Before harmonization** | | **After harmonization** | |
| --- | --- | --- | --- | --- | --- |
| **Feature class** | **Feature** | F | Imaging site | F | Imaging site |
| Intensity-based statistical features | Coefficient of dispersion |  |  |  |  |
|  | Median absolute deviation |  |  |  |  |
| Neighbourhood grey tone difference based features | Complexity |  | 0.012 |  |  |
| Morphological features | PCA elongation |  | 0.004 |  |  |
|  | Diameter |  |  |  |  |
|  | PCA flatness |  |  |  |  |
|  | PCA major axis |  | 0.021 |  |  |
| Grey level size zone based features | Zone size entropy | 25.16 | <0.001 |  |  |
| Grey level co-occurrence based features | Joint maximum | 27.09 | <0.001 |  |  |
|  | Contrast | 16.30 | <0.001 |  |  |
|  | Energy | 42.94 | <0.001 |  |  |
| Grey level distance zone based features | Zone distance non uniformity | 35.42 | <0.001 |  |  |
| Intensity histogram features | Median |  |  |  |  |
| Grey level run length based features | Run length non uniformity | 77.32 | <0.001 |  |  |
| Neighbouring grey level dependence based features | Low dependence low grey level emphasis |  | 0.006 |  |  |
